# Chronic dosing with metformin plus bosentan decreases in vitro pulmonary artery contraction from isolated arteries in adults with pulmonary hypertension

**DOI:** 10.15171/jcvtr.2019.32

**Published:** 2019-08-26

**Authors:** Shutan Liao, Dongsheng Li, Zheng Hui, Craig S McLachlan, Yang Zhang

**Affiliations:** ^1^Rural Clinical School, University of New South Wales, Sydney, New South Wales, Australia; ^2^The First Affiliated Hospital of Sun Yat-sen University, Guangzhou, China; ^3^The First Affiliated Hospital of Nanchang University, Nanchang, China

**Keywords:** Bosentan, Metformin, Pulmonary Arterial Hypertension, Congenital Heart Defect, Vessel Reactivity

## Abstract

***Introduction:*** Pulmonary arterial hypertension (PAH) specific drug therapy using bosentan has significantly improved quality of life and survival, although PAH is still an incurable disease. Recent studies suggest metformin may have additional treatment benefits in PAH. We therefore investigated in vitro pulmonary artery reactivity after combination therapy of bosentan and metformin in PAH patients as compared with bosentan monotherapy in a prospective, randomized study.

***Methods:*** Adult patients with PAH associated with congenital heart defects (PAH-CHD) were randomised to receive bosentan (initially at 62.5 mg twice daily for 4 weeks and then 125 mg twice daily) for 3 months with or without the combination treatment of metformin (500 mg twice daily). Vessel reactivity of isolated pulmonary arteries was examined using a wire myograph.

***Results:*** Phenylephrine (PE)-induced contractions of arteries in patients received combination therapy were significantly attenuated at concentrations of 3 × 10^-7^ M, 10^-6^ M and 3 × 10^-6^ M, compared to those received bosentan monotherapy. After denudation, PE-induced contractions at concentrations of 3 × 10^-6^ M and 10^-5^ M were significantly decreased in the combination therapy group. AMP-activated protein kinase (AMPK) inhibitor compound C abrogated the inhibitory effects of metformin on PE-induced contractility. AMPK and eNOS phosphorylation in the pulmonary arteries of patients treated with combination therapy was increased compared to monotherapy (*P * < 0.05).

***Conclusion:*** Adding metformin to bosentan therapy in patients with PAH-CHD decreased *in vitro* pulmonary artery contraction induced by PE, which is possibly related to increased AMPK phosphorylation.

## Introduction

Pulmonary arterial hypertension (PAH) with increased pulmonary vascular resistance (PVR) is a common complication of congenital heart defects (CHD).^1^ Over 7% of CHD patients have possible PAH and approximately 100 per million general adult population suffer from this disease.^2^ The development of PAH in CHD patients leads to markedly increased morbidity and mortality.^3,4^ Currently, there are no curative treatments for PAH associated with CHD (PAH-CHD) other than heart–lung transplantation. Therefore, management focus on slowing down the PAH progress and improving quality of life and heart function before transplantation proceeds. Selected patients may benefit from shunt closure with correctable defects, although this approach is still questionable.^5^ With the advent of PAH-specific drugs, in particular bosentan-an dual endothelin receptor (ER) antagonist that targets the endothelin (ET) pathway and stimulates vascular smooth muscle cell (VSMC) relaxation, the “treat and repair approach” has been proposed to re-evaluate the operability in PAH-CHD patients, previously thought to be unsuitable candidates for surgery.^6^ Pulmonary reactivity response to vasodilators, namely acute vasoreactivity testing, remains one of the gold standards to assess prognosis and indication for specific PAH therapy, and to assess the operability of PAH-CHD, given the existence of other more readily attainable prognostic factors.^7^

Multiple mechanistic pathways, including the ET, nitric oxide (NO) and prostacyclin pathways, are involved in the pathogenesis of PAH. These pathways promote vascular constriction of pulmonary arteries and hence increase vascular resistance and pressure. Combining drugs that target more than one pathway is an attractive option for treatment as additional benefits compared with monotherapy had been observed in previous studies.^8-10^ As a well-known antihyperglycemic agent, metformin improves insulin resistance in type 2 diabetes, and has been shown to reduce cardiovascular disease risks in those patients.^11^ Animal studies reveal that metformin reverses the development of experimental PAH in rats,^12,13^ suggesting that metformin may have additional treatment benefits in PAHs. Indeed, metformin increases the NO concentration and endothelial NO synthase (eNOS) expression and reduces the ET-1 concentration in the insulin-resistant human endothelial cells.^14^ Through AMP-activated protein kinase (AMPK), metformin not only promotes phosphorylation and activation of eNOS,^15,16^ but also decreases cytotoxic peroxynitrite, a well-known vasoconstrictor, accompanied by enhanced NO release and reduced nitroxidative stress in obese rats.^17^ Moreover, metformin-induced AMPK activation suppresses rat VSMC contraction,^18^ as wells as exaggerates phenylephrine (PE)-induced AMPK phosphorylation and attenuates contractile response in endothelium-denuded rat aorta.^19^ Song et al also demonstrated that the activity of AMPK negatively suppresses pulmonary VSMC proliferation and has possible utility in modulating pulmonary vascular remodelling.^20^

We previously found that combination therapy with bosentan and metformin in PAH-CHD patients provides additional improvements in important outcomes such as exercise capacity and pulmonary hemodynamics, compared with bosentan alone.^21^ Therefore, the current study aimed to investigate the vessel reactivity (induced constriction) of isolated pulmonary arteries from PAH-CHD patients after combination therapy of bosentan and metformin as compared with bosentan monotherapy and explore the possible mechanism of action of metformin in those patients.

## Material and Methods

In accordance with the Declaration of Helsinki, all study protocols were approved by the Institutional ethics committee of Nanchang University on human research, and written informed consent was obtained from all patients for the use of their tissue. Between May 2016 and December 2017, 93 adult patients (18-65 years) firstly diagnosed with PAH-CHD at our hospital were enrolled. The inclusion and exclusion criteria have been described previously.^21^

### Treatment

All enrolled patients were randomised to receive bosentan (initially at 62.5 mg twice daily for 4 weeks and then 125 mg twice daily) for 3 months with or without the combination treatment of metformin (500 mg twice daily). At 3 months follow-up, right heart catheterization and acute vasoreactivity testing were performed to assess the eligibility for shunt closure.^22,23^ Selected patients, assessed by our heart surgery team, underwent operations with biventricular circulation.

### Wire myography

Lung tissue biopsy was taken from the left upper lobe before cardiopulmonary bypass intraoperatively and placed in ice-cold physiological saline solution (PSS; mmol/L; 119 NaCl, 25 NaHCO3, 4.69 KCl, 2.4 MgSO4, 1.6 CaCl2, 1.18 KH2PO4, 5 glucose, 0.034 EDTA; pH 7.4). The small pulmonary arteries (internal diameter ≈250 µm) were dissected and carefully cleaned of adherent connective tissue and cut into 2 mm segments. 16 artery segments (8 of them had mechanical removal of endothelium using the steel wire) from each patient were mounted onto an isometric wire myograph system (610M wire myography; Danish Myotechniques, Aarhaus, Denmark). The vessels were bathed in PSS, gassed with 5% CO_2_/95% air, maintained at a temperature of 37°C, and allowed to equilibrate for 30 min before normalization to an internal diameter of 0.9 of L3.67kPa using normalization software (Myodata, Danish Myotechnologies, Aarhus, Denmark), which has been shown to be optimal in our preliminary study using the method described previously.^24^ Vessel viability was assessed by exposure to high-potassium PSS (KPSS, mmol/L;12.45 NaCl, 25 NaHCO3, 120 KCl, 2.4 MgSO4, 1.6 CaCl2, 1.18 KH2PO4, 5 glucose, 0.034 EDTA; pH 7.4), followed, after washout with PSS, by 1 µM PE. The constriction by PE was allowed to plateau, then 1 μM the endothelial dependent vasodilator acetylcholine (ACh) was added to test the integrity of the endothelium or ensuring complete denudation of artery rings as described previously.^25^ A concentration-response curve to PE or ET1 (0.1 nM to 10 μM) was performed. A NOS inhibitor, N^ω^-nitro-L-arginine methyl ester (L-NAME; Abcam, UK; 40 µM), was used to blocking NOS in the endothelium. An AMPK inhibitor, compound C (Abcam, UK; 40 µM), was used to test the effect of AMPK activation by metformin on vessel tone.^19^ Arteries were preincubated with either L-NAME or compound C for 30 min before a second concentration–response curve was performed. Contractile responses to PE or ET1 are shown as a percentage of the contraction induced by KPSS.

### Western blotting

Whole-cell lysates were made from homogenized isolated pulmonary artery samples with radio-immunoprecipitation assay buffer (Abcam, UK) containing a protease inhibitor cocktail (Roche, UK). Protein concentrations were determined through a Direct Detect Spectrometer (Merck, Germany). 20 µg protein from each sample was resolved by SDS-PAGE and transferred to nitrocellulose membranes for Western blotting with antibodies specifically recognizing human AMPK and phosphorylated AMPKα (p-AMPK) (Cell Signaling Technology, US), as well as eNOS, phosphorylated eNOS (p-eNOS), ERA, ERB and GAPDH (Abcam, UK). Immune complexes were visualized using horseradish peroxidase-conjugated secondary antibody with enhanced chemiluminescence on a BioRad Chemidoc MP system.

### Statistical analysis

Data are expressed as means ± SEM and were compared using one-way ANOVA or two-way repeated-measures ANOVA with Bonferroni post hoc testing where appropriate. Sigmoidal curve fitting was performed on wire myography concentration-response curve data using GraphPad Prism software 7.0 (San Diego, CA). A p-value of < 0.05 was accepted as statistically significant.

## Results

After 3 months of treatments, 20 of 28 patients with bosentan monotherapy and 18 of 24 patients with combination therapy were eligible for operations with biventricular circulation. 7 patients with monotherapy and 3 patients with combination therapy were excluded from the statistical analysis due to either unsuccessful dissection of suitable pulmonary arteries or poor response of isolated arteries to vessel constrictor or dilator.

### Adding metformin to bosentan therapy attenuates in vitro PE-induced contractility in pulmonary arteries from PAH-CHD patients

**Figure 1:**
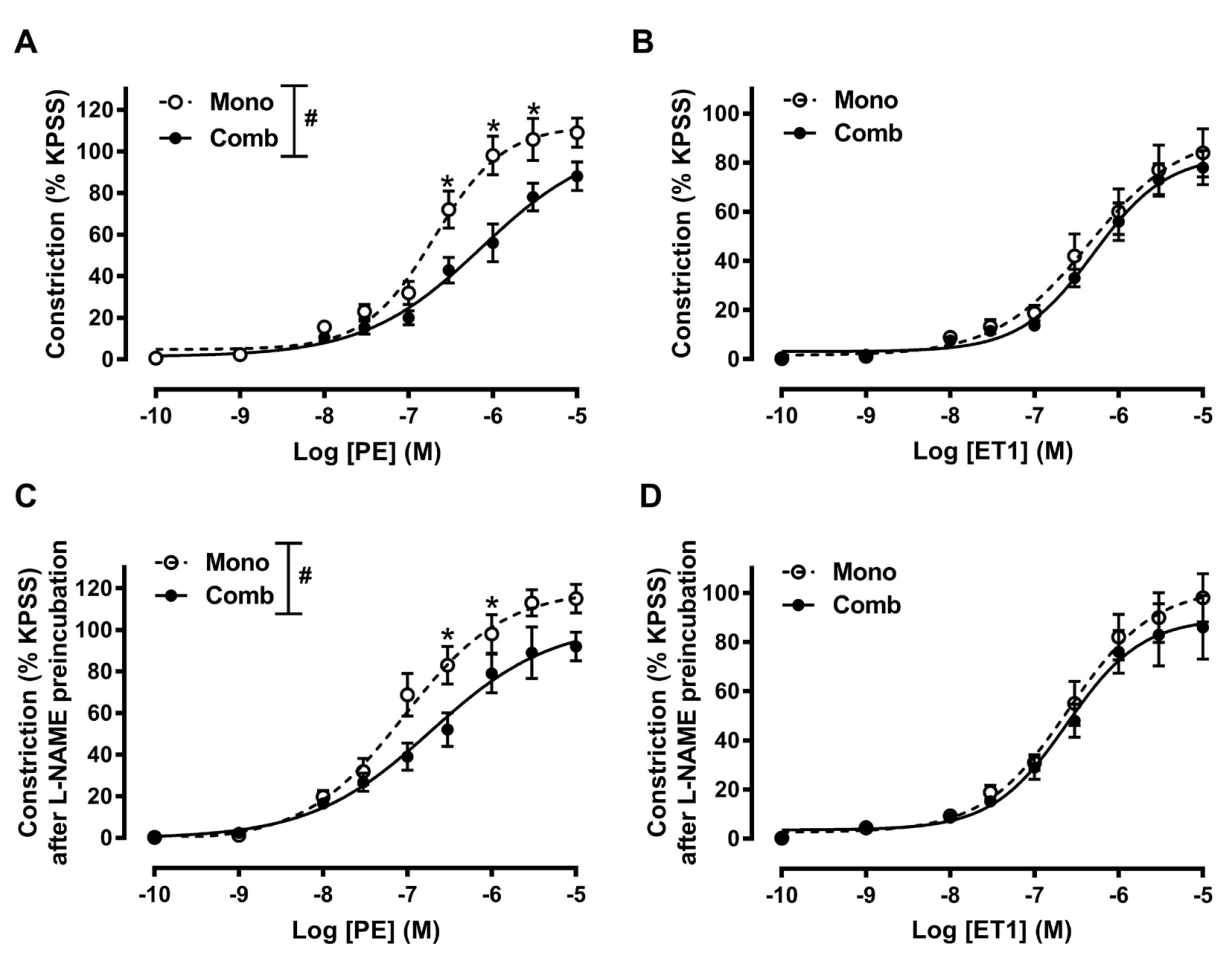
The reactivity of pulmonary artery with intact endothelium after bosentan monotherapy and bosentan/metformin combination therapy for 3 months.** (**A) and (B) Concentration-response curves for PE or ET-1 induced constriction of isolated pulmonary arteries.** (**C) and (D) Concentration-response curves for PE or ET-1 induced constriction of isolated pulmonary arteries after 30 min L-NAME preincubation. Constriction of pulmonary arteries in response to PE was significantly lower in combination therapy compared with monotherapy before or after L-NAME preincubation. Data are expressed as means ± SEM. Mono, bosentan monotherapy, n = 13; Comb, bosentan/metformin combination therapy, n = 15; ^#^ P < 0.05; two-way ANOVA; * P < 0.05; two-way ANOVA with Bonferroni post hoc test.

Both PE and ET1 induced dose-dependent constrictions in isolated pulmonary arteries with intact endothelium. These arteries were examined following prior treatments with either bosentan monotherapy or bosentan with metformin as combination therapy (Figure 1A and B). PE-induced contractions of arteries in patients received combination therapy were significantly attenuated at concentrations of 3 × 10^-7^ M, 10^-6^ M and 3 × 10^-6^ M, compared to those received monotherapy (Figure 1A). Combination treatment had no effects on ET1-induced contractions (Figure 1B).

To determine the potential mechanisms of metformin-improved NO production on pulmonary artery response, vessels were preincubated with L-NAME for 30 min to block the endothelial NOS activity. We found that L-NAME preincubation increased the contractions to PE or ET1 by decreasing the NO bioavailability. However, blocking eNOS by L-NAME did not alter differential responses to PE between arteries in patients with monotherapy or combination therapy (Figure 1C).

### Compound C prevents the inhibitory effect of metformin on PE-induced contractility

**Figure 2:**
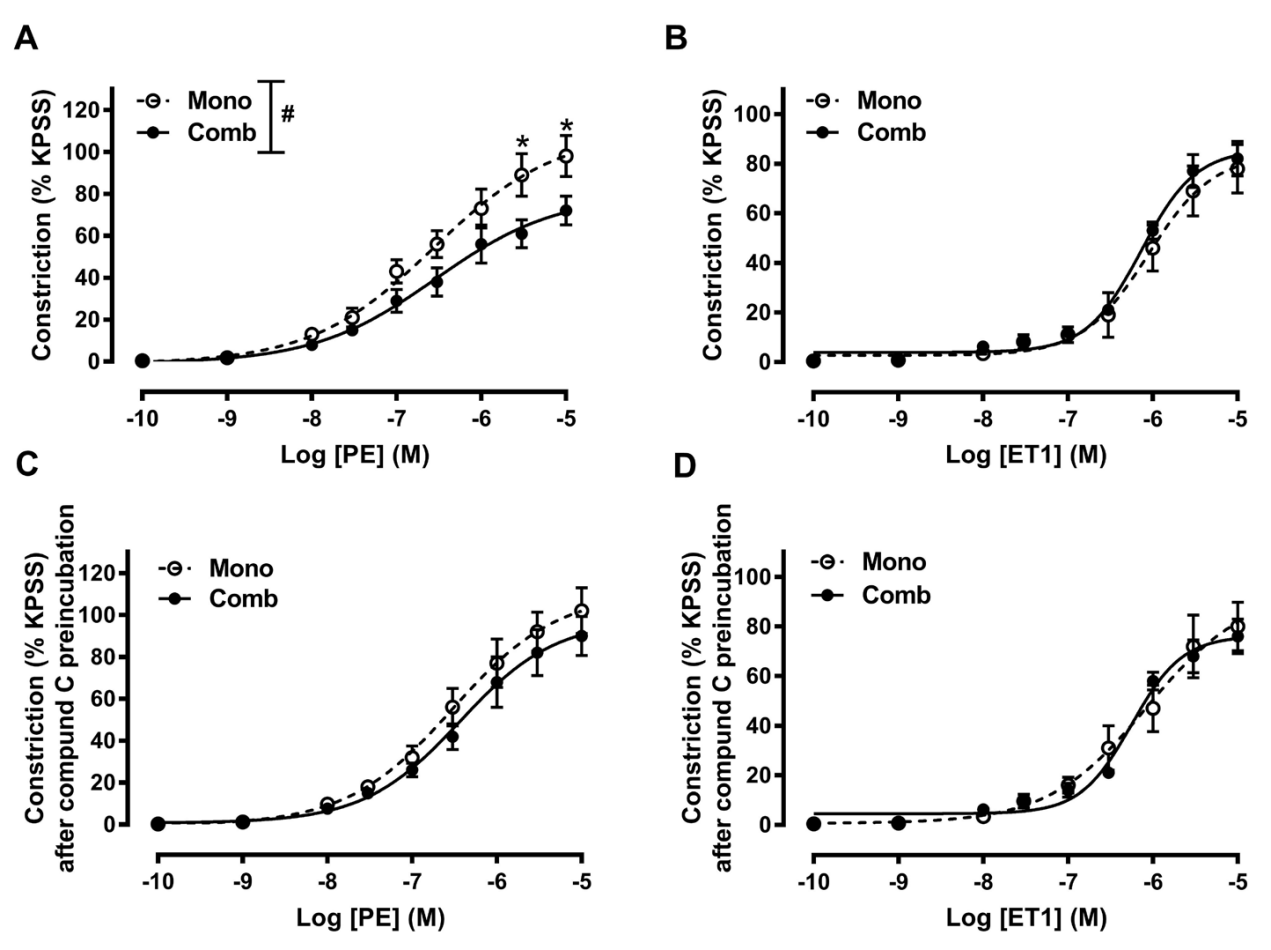
The reactivity of endothelium-denuded pulmonary artery after bosentan monotherapy and bosentan/metformin combination therapy for 3 months.** (**A) and (B) Concentration-response curves for PE or ET-1 induced constriction of isolated pulmonary arteries.** (**C) and (D) Concentration-response curves for PE or ET-1 induced constriction of isolated pulmonary arteries after 30 min compound C preincubation. Constriction of pulmonary arteries in response to PE was significantly lower in combination therapy compared with monotherapy before compound C preincubation. Compound C abrogated the inhibitory effect of metformin on PE induced contractility. Data are expressed as means ± SEM. Mono, bosentan monotherapy n = 13; Comb, bosentan/metformin combination therapy, n = 15; ^#^*P* < 0.05; two-way ANOVA; * *P* < 0.05; two-way ANOVA with Bonferroni post hoc test.

To determine the direct effect of metformin on VSMCs, we tested the vascular response to vessel constrictors on endothelium-denuded pulmonary arteries. Both PE and ET1 induced dose-dependent constrictions on endothelium-denuded pulmonary arteries (Figure 2). After denudation, PE-induced pulmonary artery contractions at concentrations of 3 × 10^-6^ M and 10^-5^ M were significantly decreased in the combination therapy group, compared to the monotherapy groups (Figure 2A). There were no significant differences in ET1-induced contractions (Figure 2B). Interestingly, preincubating the rings with compound C abrogated the inhibitory effect of metformin on PE induced contractility (Figure 2C).

### Increased AMPK and eNOS phosphorylation after adding metformin to bosentan therapy

**Figure 3:**
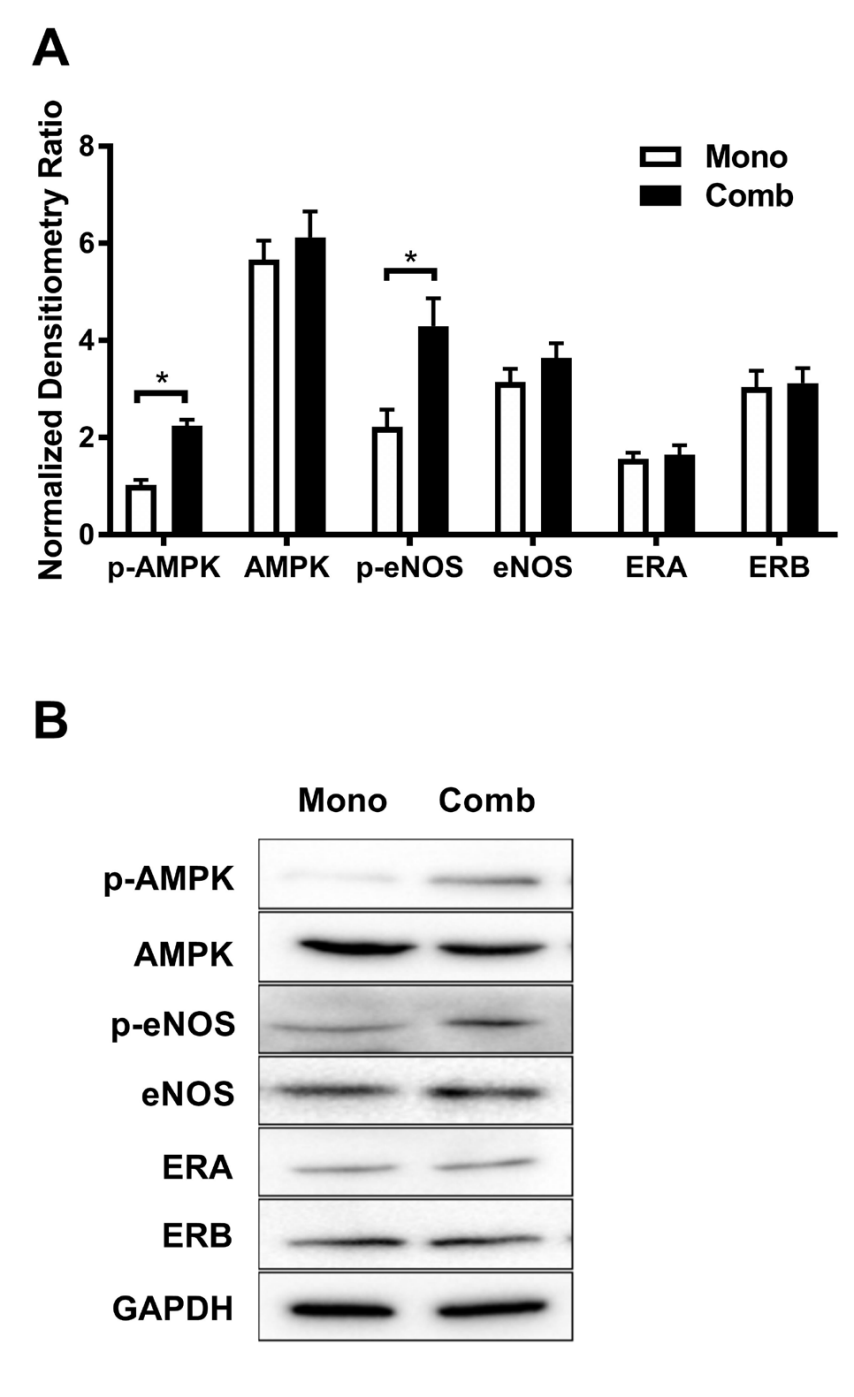
Pulmonary artery protein expressions after bosentan monotherapy and bosentan/metformin combination therapy for 3 months.** (**A**)** Protein levels of p**-**AMPK, AMPK, p-eNOS, eNOS, ERA and ERB. There were increased levels of p-AMPK and p-eNOS in the pulmonary arteries of patients treated with combination therapy, compared to bosentan monotherapy. (B) representative immunoblots of the corresponding proteins. Data are expressed as means ± SEM. Mono, bosentan monotherapy; Comb, bosentan/metformin combination therapy; * P < 0.05; one-way ANOVA.

To confirm the activity of AMPK and eNOS after metformin treatment, western blotting was performed. Western blotting revealed that increased levels of p-AMPK and p-eNOS in the pulmonary arteries of patients treated with combination therapy, compared to bosentan monotherapy (Figure 3A and B). However, there were no significant differences in the pulmonary protein levels of AMPK, eNOS, ERA and ERB between the two treatment regimens (Figure 3A and B).

## Discussion

In the present study, we compared the pulmonary artery response after 3 months treatment of bosentan monotherapy vs combination bosentan/metformin therapy and found that pulmonary artery contraction induced by PE was decreased after adding metformin to bosentan therapy in PAH-CHD patients. This is significant because PAH is characterized by progressive inflammation and vessel wall remodelling leading to increased vasoconstriction and increased pulmonary artery resistance.^26,27^

The beneficial effects of metformin on the vascular function has been suggested in clinical observations,^28^ although the molecular mechanisms are still unclear. In rats, oral administration of metformin diminished vascular reactivity to catecholamine constrictor both with and without the endothelium.^29,30^ In humans, metformin improves vascular function in patients presented with insulin resistance.^31,32^ We found that increased phosphorylation of AMPK and eNOS after chronic metformin treatment, which is consistent with results from previous translational studies with metformin treatments.^33-35^ The AMPK-eNOS-NO pathway is therefore considered to be the main pathway attributing to the regulative effect of metformin on vascular function.^36^ AMPK is a heterotrimetric enzyme comprising a catalytic subunit and two regulatory subunits. The catalytic subunit consists of an N-terminalcatalytic kinase domain and a C-terminal regulatory domain. The phosphorylation of the kinase domain by upstream kinases is required for AMPK activation.^37^ AMPK not only is involved in the regulation of cellular and organ metabolism,^38^ but also plays a regulatory role over vascular structure and function and is essential for the maintenance of cardiovascular health.^39^ Activation of AMPK by metformin stimulates NO synthesis in vascular endothelial cells by increasing phosphorylation and activation of eNOS^15,40^ and reduces mitochondrial reactive oxygen species, which diminish the bioavailability of NO.^41^ However, blocking the eNOS or removing the endothelium did not fully reverse the inhibitory effect on vessel constriction by metformin in our study. A direct effect of metformin on VSMCs is suspected. Indeed, it is reported that the activation of AMPK by metformin suppresses VSMC contraction by inhibiting myosin light chain (MLC) kinases and MLC phosphorylation in rats,^18^ reduces VSMC proliferation and migration,^42^ and attenuates elevation of intracellular Ca^2+^ levels in VSMCs.^43,44^ A direct effect via AMPK on smooth muscle cell wall of the artery is supported in our study, where we found that the AMPK inhibitor compound C reversed the inhibitory effect on PE-induced contraction by metformin on endothelium-denuded pulmonary arteries. Consistent with this, metformin increases AMPK phosphorylation and attenuates contractile responses in endothelium-denuded rat aorta,^19^ suggesting a potential role of AMPK as an intermediary signalling component for metformin action in pulmonary artery response.

It is worthy to note that compound C is the primary reagent used as an AMPK inhibitor and has been widely used in biochemical *in vitro* and some *in vivo* experiments. However, Compound C unfortunately inhibits several other kinases much more potently than AMPK and is therefore highly non-specific, and its inhibitory effects seem to be dose-dependent.^45,46^ Furthermore, ET1 is a strong vasoconstrictor and is one of key mediators of PAH development.^47^ It is reported that the activation of AMPK by metformin suppresses ET1 induced pulmonary artery VSMC proliferation in rats,^48^ and inhibits *ET1* expression at the transcriptional and translational level in the aorta.^49^ Additional effects of metformin on the ET pathway and/or other pathways may also contribute to the differential vascular responses we observed.

## Conclusions

In summary, our findings suggest that adding metformin to bosentan therapy in patients with PAH-CHD decreased *in vitro* pulmonary artery contraction induced by PE, which is possibly related to increased AMPK phosphorylation. Our findings demonstrate the efficacy of metformin clinically in diseased pulmonary arteries.

## Competing interests

The authors report no relationships that could be construed as a conflict of interest.

## Ethical approval

In accordance with the Declaration of Helsinki, all study protocols were approved by the Institutional ethics committee of Nanchang University on human research, and written informed consent was obtained from all patients for the use of their tissue.

## Acknowledgments

The authors gratefully acknowledge the financial support from The Limingzhang Sciences Foundation (2014-778903). Shutan Liao is funded under an NHMRC Development Grant.
